# Case of a Large and Several Smaller Encysted Tumors in the Right Ovarium, Being Discharged through an Opening in the Abdominal Parietes, Consequent on Suppurative Inflammation

**Published:** 1822-06

**Authors:** 


### OBSTETRICAL MEDICINE.

**Art. VI.—*Case of a large and several smaller Encysted Tumors in the right Ovarium, being discharged through an Opening in the Abdominal Parietes, consequent on Suppurative Inflammation.* By the Editor.**

ELIZABETH KENT, aged 35, the mother of four children, presented herself for admission at the Westminster General Dispensary, on the 3d of September, under the following circumstances:—She was in the fourth month of pregnancy, and had two large swellings in the abdomen, hard, irregular, and very distinct from the general enlargement of that cavity peculiar to her situation. She suffered greatly from pain in the back and groins; was constantly feverish and heated; and felt, otherwise, very uncomfortable. On examining more particularly the region of the abdomen, I found a moveable tumor, of the size of a child's head, in the right hypochondrium, resting, apparently, on the fossa iliaca of that side. This tumour, as far as the advanced state of pregnancy would allow, could be shifted several inches higher than, or on one side of, its usual position. It appeared as if situated in the right ovarium, and was, probably, formed by one of the cells of that organ preternaturally enlarged. The patient assured me she had had this swelling, together with another in the left side, of less magnitude but harder than the former, and immoveable, for two or three years; and had been told by the midwife and medical attendant during her last confinement, that it had materially contributed to the difficulty of her labour on that occasion, by descending into the excavation of the pelvis simultaneously with the head of the child. Such had been her sufferings on that occasion, owing to the morbid derangement of the parts, that the poor woman dreaded the approach of her present confinement, from an apprehension lest the tumor, now much larger than before, should prove a fatal obstacle to her delivery.

I first endeavoured to quiet her fears with a few words of explanation as to the nature of her complaint; and subsequently promised to attend her in person, should she meet with any difficulty, or feel a wish that I should assist her in her confinement.

As the period of pregnancy advanced, the patient became still more restless, feverish, and uncomfortable, so as twice to require the abstraction of blood, and the administration of several medicines; to enumerate which would be now out of place. On the approach of the conclusion of her term, she was suddenly seized with a most distressing sensation of bearing down, accompanied by a decided depression of the abdominal swelling, as if the child had descended into the pelvic cavity. On examination, I found that the head of the child rested on the os uteri, which it partially distended; and, on gently pushing my index between the shortened neck of the uterus and the right and upper side of the vagina, I could distinctly perceive the *moveable* tumor on a level with the child's head.

Although upwards of three weeks were yet wanting to complete the full period of gestation, I felt that, unless some measure were adopted to check the farther descent of the tumor, or even to dislodge it from its present situation, considerable obstacle would be presented by it to her safe and speedy delivery; and I, therefore, bethought myself of the following scheme.

Having desired the patient to lie in a recumbent posture, and relieved the bladder of a great portion of its contents, I availed myself of the mobility of the tumor to move it gently and gradually, by means of the finger introduced into the vagina, first obliquely upwards,—guiding its progress, as it were, through the abdominal parietes with the left hand; and subsequently, pushing it with the latter towards the central region of the abdomen, under the linea alba, immediately above the bladder, and rather below the umbilicus. Here it became fixed, and I studied to keep it so by an appropriate bandage. It formed a very distinct prominence above the general swelling of the abdomen; and I trusted that its presence in this spot would not prove injurious to the child, since none but the inferior extremities of the latter could at all be subject to any pressure which the tumor might be expected to exert on the uterus. The advantage to be derived from having thus placed the moveable tumor, I took to be this,—that, as it would naturally glide (as indeed it had already glided,) down the inclined plane of the fossa iliaca, if suffered to remain with all its freedom of motion in its former position, so as hereafter to interfere materially with the labour,—here, where its attachment to the womb was put on the stretch by the change of position, and the locality it was made to occupy was much narrowed; its further movement would be effectually prevented.

I took this opportunity again of examining, externally, the tumor in the left hypochondrium, which I found rather smaller than the former, but more irregular in its surface, and perfectly immoveable. Nothing, therefore, was to be apprehended from it during parturition.

At the expiration of three weeks from this time, the patient was delivered of a male child, after a very easy and quick labour, during the whole progress of which the moveable tumor remained fixed. On the expulsion of the after-birth, and the subsequent contraction of the uterus, it was remarked that the tumor did not alter its position, although much more space existed now, in the cavity of the abdomen, for such an evolution. When the tumor was pressed with the hand, a slight pain was excited.

I visited this patient for some days afterwards, keeping my attention directed to the tumor; but she proceeded so favourably through her puerperal period, that I discontinued my attendance.

Two or three weeks after she had been discharged from the register of the Dispensary, in good health, and suckling her baby, likewise healthy; an application was made to me to visit her at her lodgings, in consequence of an increase in the original swelling, or tumor, attended with considerable pain. On examining the part, I found it red, acuminated, throbbing, and presenting slight indications of fluctuation. I ordered a linseed-meal poultice to be applied warm, immediately, and to be frequently repeated. At the expiration of two days the swelling broke, and a large quantity of white thick pus flowed from the opening, followed by a cyst composed of a dense white membrane of great consistency, which, when filled with water, might be compared, for size, to the head of a full-grown fetus, having an opening of the same diameter of that formed in the parietes of the abdomen, through which it had escaped.

As I had been informed by the husband when first summoned to her that the tumor had broke, I requested the attendance of my friend and colleague, Mr. A. C. Hutchison, one of the surgeons of the Dispensary, who assisted in extracting the capsule in question already partially emerged, and who with myself and several other practitioners to whom the preparation has been shown, consider the case as singular and interesting. Several smaller capsules followed the larger one, of a brownish colour, dense and intact, containing no fluid whatever, and presenting none of the characters of hydatids.

From this moment the wound began to heal; and, although much hectic fever was present during the period of suppuration, bark and an improved diet soon overcame this symptom, and the woman was ultimately restored to comparative health.

There are now scarcely any traces of the former swelling; but the part is tender to the touch, and the abdomen appears generally enlarged.

The cysts are now preserved in my collection at the Westminster General Dispensary, for the inspection of any member of the profession who may be inclined to investigate the case, and examine the patient.

The many inferences which naturally suggest themselves on this occasion, I will gladly pass over in silence; as I neither wish to forestal the conclusions which persons versed in this branch of practice might form, on a perusal of a case; nor am I disposed to come to any conclusion myself, on the evidence of a single fact, however extraordinary. A. B. G.

